# Hydrogen Sulfide Biomedical Research in China—20 Years of Hindsight

**DOI:** 10.3390/antiox11112136

**Published:** 2022-10-28

**Authors:** Rui Wang, Chaoshu Tang

**Affiliations:** 1 Department of Biology, Faculty of Science, York University, Toronto, ON M3J 1P3, Canada; 2 Department of Physiology and Pathophysiology, Peking University Health Science Centre, Beijing 100191, China

**Keywords:** angiogenesis, atherosclerosis, chronic obstructive pulmonary disease (COPD), gasotransmitter, heart failure, hydrogen sulfide, hypertension, pain, reproduction, therapeutic

## Abstract

Hydrogen sulfide (H_2_S) is an important gasotransmitter that is produced by mammalian cells and performs profound physiological and pathophysiological functions. Biomedical research on H_2_S metabolism and function in China began 20 years ago, which pioneered the examination of the correlation of abnormal H_2_S metabolism and cardiovascular diseases. Over the last two decades, research teams in China have made numerous breakthrough discoveries on the effects of H_2_S metabolism on hypertension, atherosclerosis, pulmonary hypertension, shock, angiogenesis, chronic obstructive pulmonary disease, pain, iron homeostasis, and testicle function, to name a few. These research developments, carried by numerous research teams all over China, build nationwide research network and advance both laboratory study and clinical applications. An integrated and collaborative research strategy would further promote and sustain H_2_S biomedical research in China and in the world.

## 1. Introduction

One of the worst industrial accidents in China killed about 243 people and hospitalized 2142 due to the leakage of poisonous gases from one natural gas well in the Northeast gas field, turning a 25-square-kilometer area into a “death zone” in southwest China [1]. The fatal poison in the natural gas was hydrogen sulfide (H_2_S). The year was 2003.

The lethal nature of H_2_S gas is remnant of that of nitric oxide (NO) and carbon monoxide (CO). All of these three gas molecules had been categorized as gasotransmitters for their capability of mediating physiological functions in mammalian cells [2].

H_2_S is produced in almost all types of mammalian cells. Using L-cysteine or homocysteine as the substrates, cystathionine gamma-lyase (CSE), cystathionine beta-synthase (CBS), and 3-mercaptopyruvate sulfurtransferase (MST) catalyze the enzymatic production of H_2_S and other metabolic products such as ammonia and pyruvate [3]. Non-enzymatic production of H_2_S in eukaryocytes can be realized through the reaction of cysteine with iron and vitamin B6 [4]. Reduction in elemental sulfur using NADPH or glucose oxidation in phospho-gluconate pathway [5] also produce H_2_S non-enzymatically.

Inspired by the potential gasotransmitter role of H_2_S and intrigued by its “Jekyll and Hyde” dual identities, a research team led by Drs. Tang C and Du J from Peking University, China reported a series of observations on the correlation of H_2_S with an array of cardiovascular diseases. They found that during septic shock and endotoxin shock the arterial H_2_S content was significantly higher in the experimental rats [6]. On the other hand, endogenous H_2_S levels were lower in the blood as well as in aortic and superior mesenteric artery tissues in spontaneously hypertensive rats, which was likely due to the inhibition of nitric oxide synthase [7]. In experimental rats with hypoxic pulmonary hypertension, H_2_S level was also lowered than those without pulmonary hypertensive rats [8]. These reports in 2003, together with a preceding short communication describing H_2_S as a messenger molecule in cardiovascular system in early 2002 [9], marked the beginning of H_2_S biology and medicine research in China. Over the last 2 decades, scientific exploration of the biomedical importance of H_2_S has blossomed in China, which leads the global trend in many areas. These research developments in China have contributed significantly to the acknowledgement and acceptance of the dual identities of H_2_S as being a lethal toxic gas and a life saver [10].

## 2. Three Phases of H_2_S Research in China

**Figure 1:**
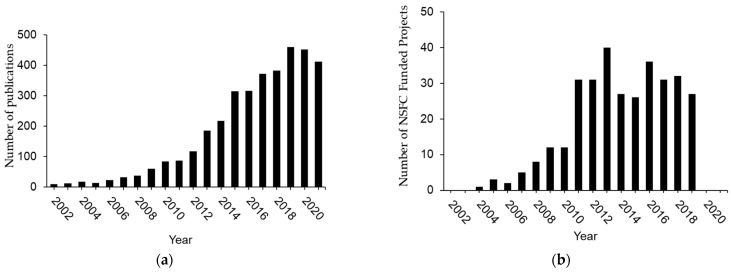
Trends and intensities of biomedical research on H_2_S in China. (**a**) The numbers of publications on H_2_S biology and medicine by Chinese researchers. (**b**) The NSFC-sponsored research projects on biomedical research of H_2_S held in China.

H_2_S-focused scientific exploration in China from 2002–2007 opened new frontiers of biomedical research not only in China but also for the world in various fields. During this first phase of investigation, majority of the research activities was conducted by Tang and his collaborators in Peking University, Beijing. The research teams led by Drs. Zhu YC and Zhu YZ also started to publish their results on the cardiovascular effects of H_2_S with Fudan University as the affiliation institute [11,12,13]. The national focus of H_2_S research was on the cardiovascular effects of H_2_S and the underlying mechanisms. More than 65 peer-reviewed scientific papers appeared on international journals with Chinese universities as the affiliated institutes (Figure 1a). During this period, about 10 research projects on H_2_S biology and medicine were supported by National Natural Science Function of China (NSFC) (Figure 1b).

The pinnacle of this phase was epitomized by the 1st China National symposium on H_2_S gasotransmitter in 2007 in Beijing (Table 1). The symposium was organized by Peking University, Chinese Medical Doctor Association, and Fudan University. More than 160 Chinese delegates gathered to present their discoveries, exchanged and debated their views and ideas. Noticeable was that Hebei Medical University, Chongqing Medical University and Qinghai Medical College also communicated their H_2_S research observations.

Phase 2 of H_2_S biomedical research in China was from 2008 to 2013. The research scope was significantly enlarged beyond the role of H_2_S in the cardiovascular system. More than 221 peer-reviewed scientific papers appeared on international journals with Chinese universities as the affiliated institutes. During this period, 160 H_2_S research projects had received national funding from NSFC (Figure 1b). In 2009, “The First International Conference of H_2_S in Biology and Medicine” was held in Shanghai, China. While 6 international Conferences on H_2_S biology and medicine in total have been organized in different countries worldwide, the first one of them held in China provided an important impetus for H_2_S research in China when leading international investigators in H_2_S biology initially came together to discuss this important molecule and its effects on cellular redox and chemical biology, signal transduction, and pathophysiological functions under healthy or disease conditions. The 2nd China national symposium on H_2_S gasotransmitter was held in 2010 in Beijing (Table 1). There were 150 representatives from more than 40 research institutes in 13 provinces and cities. During the symposium, 77 presentations covered the metabolism and biological functions of H_2_S from the angles of physiology, pathophysiology, pharmacology and pharmacy as well as clinical applications. With the support of NSFC, the 3rd China national symposium on H_2_S gasotransmitter was held in Beijing with more than 200 participants (Table 1). This symposium further engaged numerous Chinese research teams in the fundamental and clinical settings with the focus on H_2_S metabolism and functions.

Phase 3 of Chinese H_2_S biomedical research was from 2014 to now. This current phase is featured by the depth and width of H_2_S research in China and its global impact. About 3000 peer-reviewed scientific papers appeared on international journals with Chinese universities as the affiliated institutes (Figure 1a). During this period, NSFC supported 185 research projects on H_2_S biology and medicine (Figure 1b).

The authors of this review searched the database from Clarivate’s Web of Science^TM^ –Core Collection with the combination of the topic of “Hydrogen sulfide” or “H_2_S”. In order to retrieve publication data pertinent closely to Biology and Medicine, the initial results were refined with the following Web of Science^TM^ Categories chosen: Biochemistry Molecular Biology; Chemistry Analytical; Cell Biology, Pharmacology Pharmacy; Biotechnology Applied Microbiology; Microbiology; Food Science Technology; Physiology; Medical Science Experimental; Neuroscience; Biology; Peripheral Vascular Disease; Cardiac Cardiovascular Systems; Marine Freshwater Biology; Chemistry Medicinal; Gastroenterology Hepatology; Spectroscopy; Biophysics; Biochemical Research Methods; Critical Care Medicine; Medicine General Internal; Urology Nephrology; Nutritional Dietetics; Hematology; Genetics Heredity; Dentistry Oral Surgery Medicine; Clinical Neurology; Zoology; Veterinary Sciences; Engineering Biomedical; Pathology; Ophthalmology; Evolutionary Biology; Geriatrics Gerontology; Obstetrics Gynecology; Reproductive Biology; Pediatrics; Anesthesiology; Medicine Legal; Behavioral Sciences; Integrative Complementary Medicine; Medical Laboratory Technology; Rheumatology; Psychiatry; Paleontology; Infectious Diseases; Developmental Biology; Sport Sciences; Radiology Nuclear Medicine Medical Imaging; Dermatology; Emergency Medicine; Cell Tissue Engineering; Mathematical Computational Biology; Imaging Science Photographic Technology; Rehabilitation; Virology; Psychology Biological; Allergy; Tropical Medicine; Entomology; Parasitology; Orthopedics; Anatomy Morphology; Psychology; Health Care Sciences Services; Microscopy; Psychology Experimental; Gerontology; Neuroimaging; Nursing; Primary Health Care; Psychology Multidisciplinary; Psychology Developmental.

**Figure 2:**
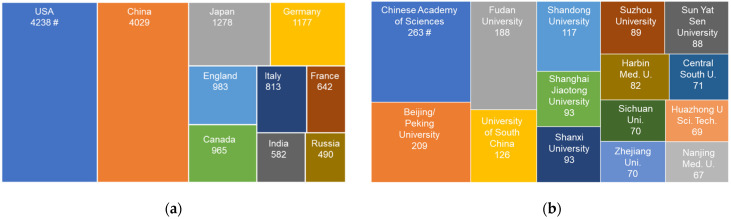
Global and regional distributions of the research publications on H_2_S biology and medicine. (**a**) Top 10 countries with the most H_2_S biomedical research publications. (**b**) Top 20 Institutional affiliations of H_2_S biomedical research publications in China.

**Figure 3:**
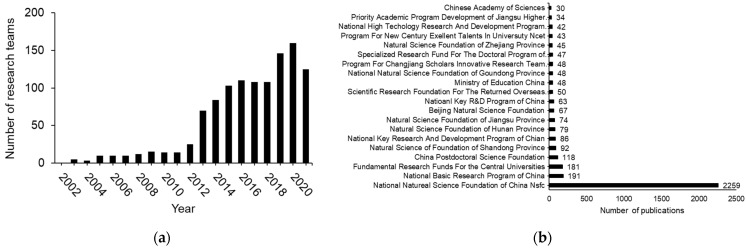
Chinese national research teams and funding agencies for H_2_S biomedical research. (**a**) Active H_2_S biomedical research teams in China. (**b**) Top 20 research funding agencies and authorities in China who sponsored H_2_S biomedical research publications from Chinese research teams.

A total of 18,017 publications on H_2_S biology and medicine over all time were counted worldwide. USA has contributed the most H_2_S biomedical research papers. China is home to 2nd most H_2_S publications in this field. Japan, Germany, and UK occupy the rest spots of the top 5 countries in the world who have the most H_2_S biomedical publications (Figure 2a). We then combine the topic (hydrogen sulfide or H_2_S) and address (China) with January 2002 to December 2021 as the Publication Date. Over this span of 20 years, 3609 articles on H_2_S biology and medicine were published by Chinese research teams. According to Web of Science^TM^, about 250 Chinese institutions have publications on H_2_S biology and medicine over the last 20 years, each with at least three papers. Many of these institutions have multiple research teams committed themselves to H_2_S research. In terms of the institutions with the most H_2_S research papers published, the top 5 in China are Chinese Academy of Sciences, Beijing/Peking University, Fudan University, University of South China, Shandong University (Figure 2b). Shanghai Jiao Tong University, Shanxi University, Suzhou University, Sun Yat Sen University are also hotbeds of H_2_S research, each with than more 85 papers published. There are more than 100 active H_2_S research teams, counted per year since 2014 (Figure 3a). The most H_2_S biomedical publications by Chinese research teams have been supported financially by NSFC (Figure 3b). Numerous funding agencies and authorities at regional and provincial levels also sponsored H_2_S biomedical research in China (Figure 3b).

## 3. Major Discoveries Made by H_2_S Researchers in China

### 3.1. The Correlation of H_2_S Metabolism with Cardiovascular Diseases

#### 3.1.1. Blood Pressure Regulation by H_2_S and the Underlying Mechanisms

*CSE* gene was firstly cloned and sequenced in the cardiovascular system in 2001 [14]. CSE-generated H_2_S caused vasorelaxation by stimulating K_ATP_ channels in vascular smooth muscle cells [14]. The potential correlation of H_2_S level and cardiovascular diseases, however, was unknown until Tang and his collaborators in China draw the connections in 2002. They reported low endogenous H_2_S levels in the blood as well as aortic and superior mesenteric artery tissues in spontaneously hypertensive rats (SHR), inaugurating the studies on endogenous H_2_S in cardiovascular diseases [7]. Tang’s team further induced hypertension in Wistar rats by the oral administration of NG-nitro-L-arginine methyl ester (L-NAME) for 6 weeks. These hypertensive rats exhibited decreased CSE mRNA expression and CSE activity. Administration of H_2_S salt, NaHS, to these hypertensive rats significantly lowered the systolic blood pressure by 19% [15]. In experimental rats with hypoxic pulmonary hypertension, H_2_S level was obviously lowered than those without pulmonary hypertensive rats [8]. The studies also showed that the down-regulated endogenous H_2_S pathway is involved in high-salt-induced hypertension [16,17]. On the other hand, higher endogenous H_2_S levels were observed in hypotensive rats with septic shock induced by cecal ligation and puncture of endotoxic shock induced by injection of endotoxin [6].

The research team from Peking University conducted a series of pioneer studies to examine the role of endogenous H_2_S in the development of different types of pulmonary hypertension. In animals with hypoxia-induced pulmonary hypertension, endogenous H_2_S/CSE pathway was downregulated as compared with those without hypoxia-induced pulmonary hypertension [8,18]. In monocrotaline-induced pulmonary hypertension, endogenous H_2_S pathway was also down-regulated [19]. Interestingly, in animal models of high pulmonary blood flow-induced pulmonary hypertension, endogenous H_2_S pathway was up-regulated at the early stage but down-regulated at the late stage [20]. The intervention of H_2_S pathway to keep the H_2_S concentration at its appropriate level significantly attenuated the pulmonary artery structural remodeling and pulmonary hypertension; furthermore, abnormal endogenous H_2_S pathway is a crucial mechanism for pulmonary hypertension by which H_2_S inhibited pulmonary endothelial inflammation, smooth muscle cell proliferation and collagen remodeling but facilitated the endothelial cell apoptosis via different signaling pathways [21,22].

In all these studies, endogenous H_2_S contents were negatively correlated with blood pressure and cardiac functions. Whether altered endogenous level of H_2_S was the cause or consequence of blood pressure change was unsettled. CSE protein expression in vascular smooth muscle cells was detected in 2006 [23]. The causative relationship between lowered endogenous H_2_S level and development of hypertension was firstly established in 2003 [7,15]. Later in 2008, after knocking out *cse* gene expression in the mouse, a Canada/USA team showed that CSE expression deficiency and the resulting minimization of endogenous production of H_2_S resulted in age-dependent development of hypertension due to the loss of endothelium-dependent vasorelaxation [24]. Before the study of [24], the physiological importance of H_2_S in any type of cells or organs was deduced from several lines of indirect evidence, i.e., the existence of H_2_S-producing enzymes, the measurable levels of endogenous H_2_S in the examined cells or organs, the functional outcomes of pharmacological blocking of the H_2_S-producing enzymes, and functional changes induced by exogenous H_2_S salts or donors. The 2008 study by Yang et al. is a milestone discovery that presented the first comprehensive evidence for the role of endogenous H_2_S for any systems in mammalian body.

Numerous research teams in China devoted their efforts to explore the involvement of H_2_S in various types of hypertension as well as the mechanisms of the vascular effects of H_2_S over the last two decades. For example, Yuming Wu’s team at Hebei Medical University reported low levels of plasma H_2_S and CSE expression in renal arteries from hypertensive patients and 2K1C (two-kidney, one-clip) hypertensive rats, respectively [25]. After 12 h incubation, NaHS ameliorated endothelium-dependent relaxation of renal arteries of hypertensive patients by promoting the release of NO. In the 2K1C hypertensive rats, 20-week NaHS treatment upregulated CSE expression, increased plasma H_2_S levels, normalized endothelial function and decreased blood pressure [25]. Among H_2_S-targeted proteins involved in hypertension-resulted endothelial dysfunction are thioredoxin interacting protein (TXNIP), MAPK, and eNOS [26]. H_2_S also improves endothelial dysfunction in renal hypertension by activating peroxisome proliferator activated receptor delta (PPARδ) and inhibiting BMP4/COX-1 pathways [25].

Neurogenic causes of essential hypertension have been studied extensively, in which the functionality of carotid sinus baroreflex and sympathetic outflow are important regulatory knobs. Chronic hypertension results in a compensatory shift in baroreflex activation to a higher set point. The carotid sinus baroreceptor sensitivity in SHR was decreased. NaHS perfusion of the isolated carotid sinus from SHR facilitated baroreceptor sensitivity and inhibited sympathetic outflow, contributing to the negative feedback control of blood pressure. Activation of GABAA receptors and K_ATP_ channels, and inactivation of L-type Ca^2+^ channels may explain the regulatory effect of H_2_S on baroreceptor sensitivity [27]. Endogenous H_2_S metabolism in the rostral ventrolateral medulla (RVLM) is related to sympathetic regulation of blood pressure. In the RVLM of SHR, CBS expression and H_2_S production are lower than normotensive rats [28]. Microinjection of hydroxylamine, a CBS inhibitor, into the RVLM increased renal sympathetic nerve activity, heart rate, and arterial blood pressure, which were all reversed by micro-injection of NaHS or overexpression CBS by adenovirus vectors in RVLM [28].

#### 3.1.2. H_2_S-Inhibited Vascular Smooth Muscle Proliferation

By the year 2004, we knew nothing about the effect of H_2_S on cell proliferation and cell cycle regulation. The ice breakers are Drs. Tang C and Du J and their team members who investigated the involvement of H_2_S in the proliferation of cultured rat aortic smooth muscle cells (SMCs). By measuring ^3^H-thymidine incorporation, Du et al. [29] showed that exogenous H_2_S decreased SMC proliferation whereas endothelin-1 stimulated it. This effect of H_2_S was ascribed to the inhibited mitogen-activated protein kinase (MAPK) activity in SMCs by H_2_S. This pioneering study on rat SMC was echoed by another study in the same year, in which H_2_S at physiologically relevant concentrations induced apoptosis of human aortic SMCs (HASMCs) [30]. Of the three investigated MAPKs, exogenous H_2_S treatment targeted at extracellular signal-regulated kinase (ERK), but not p38 MAPK or c-Jun N-terminal kinase, to activate caspase-3. The latter induced apoptosis of HASMCs. The totality of decreased proliferation [29] and increased apoptosis [30] of vascular SMCs in response to H_2_S would prevent the vascular remodeling, encountered in numerous vascular diseases. Continued exploration in this field revealed that H_2_S inhibited vascular SMC proliferation by persulfidating FOXO1 at Cys457 and subsequently preventing FOXO1 phosphorylation at Ser256 [22].

H_2_S exerts epigenetic control of vascular SMC proliferation by region-specific chromatin remodeling of MAPKs pathway-associated genes [31]. Brahma-related gene 1 (Brg1) is the central catalytic subunit of an ATP-dependent chromatin remodeling complex, SWI/SNF apparatus. H_2_S inhibited the transcription and expression of Brg1, based on results from a luciferase reporter assay, real-time PCR and Western blotting. A chromatin immunoprecipitation assay indicated that H_2_S inhibited the recruitment of Brg1 to the promoter regions of the proliferation-related genes, including proliferating cell nuclear antigen (Pcna), neurotrophin 3 (Ntf3), and platelet-derived growth factor subunit A (Pdgfα) in endothelin-1-stimulated proliferative vascular SMCs. Finally, the lines of evidence from overexpression and knockdown of Brg1 were presented that Brg1-based epigenetic control was crucial for H_2_S-induced inhibition of vascular SMC proliferation [31].

#### 3.1.3. H_2_S Regulation of Angiogenesis and Atherosclerosis

The hypothesis that H_2_S would promote angiogenesis was tested and confirmed for the first time in 2007 [11]. This report is among the mostly cited papers published by H_2_S research teams in China (Web of Science). With cultured vascular endothelial cells, Cai et al. showed that NaHS increased cell proliferation, adhesion, migration, scratched wound healing, and tube-like structure formation. The phosphatidylinositol 3-kinase (PI3K) inhibitor LY 294002 or transfection of a dominant-negative mutant of Akt inhibited the effects of NaHS. Akt phosphorylation was increased by NaHS but inhibited by LY 294002 or wortmannin. The in vitro effects of NaHS on endothelial proliferation were further validated by the increased neovascularization in vivo in mice after NaHS injection [11].

Another first attempt in the world by a Chinese team to correlate H_2_S metabolism with atherosclerosis development occurred in 2009 [32]. Plasma aortic tissue levels of H_2_S were decreased but that of intracellular adhesion molecule-1 (ICAM-1) increased in apolipoprotein-E knockout (apoE-KO) mice. NaHS or propargylglycine (PPG) treatments of ApoE-KO mice decreased or increased size of atherosclerotic plaque and plasma and aortic ICAM-1 levels, respectively. NaHS suppressed ICAM-1 expression in tumor necrosis factor (TNF)-alpha-treated human umbilical vein endothelial cells (HUVECs). This study suggested the anti-atherosclerotic effect of exogenous H_2_S in apoE-KO mice [32]. Another Chinese team showed the similar anti-atherosclerotic effects of exogenous H_2_S and correlation of plasma level of H_2_S with the diabetes-accelerated atherosclerosis [33]. The inhibition of oxidative stress via Keap1 sulfhydration and Nrf2 activation were ascribed as the mechanisms for H_2_S protection in these streptozotocin (STZ)-induced LDLr(−/−) mice [33]. Direct evidence for the role of endogenous H_2_S in atherosclerosis was derived from the study by Mani et al. in 2013 from Canada. In comparison with wide-type mice, CSE-knockout mice fed with atherogenic diet developed earlier fatty streak lesions in the aortic root, more elevated plasma levels of cholesterol and low-density lipoprotein cholesterol, hyperhomocysteinemia, increased lesional oxidative stress and adhesion molecule expression, and enhanced aortic intimal proliferation. Administration of NaHS to CSE-knockout mice to compensate diminished endogenous H_2_S inhibited the accelerated atherosclerosis development. It was concluded that decreased endogenous H_2_S production predisposes the animals to vascular remodeling and early development of atherosclerosis [34]. To further explore the potential mechanisms by which H_2_S inhibits OX-LDL induced the inflammatory response in atherosclerosis, the scientists from Peking University revealed that endogenous H_2_S inhibited ox-LDL-induced macrophage inflammation by suppressing NF-κB p65 phosphorylation, nuclear translocation, DNA binding activity, and recruitment to the MCP-1 promoter. The sulfhydration of free thiol group on cysteine 38 in p65 served as a molecular mechanism by which H_2_S inhibited NF-κB pathway activation in ox-LDL-induced macrophage inflammation, which might be involved in the development of atherosclerosis [35].

#### 3.1.4. The Role of H_2_S in Heart Failure and Myocardial Pathogeneses

The studies from Peking University showed that H_2_S played an important role in the protection against myocardial damage induced by hyperhomocysteinemia, and the underlying mechanisms involved the control of the endoplasmic reticulum stress in rats [36]. Myocardial hypertrophy, also called hypertrophic cardiomyopathy (HCM), is abnormal structural change in the heart muscle which eventually leads to heart failure. With HCM, left ventricular myocardial mass is increased with consequential decrease in cardiac output or arrhythmias. The applications of NaHS and GYY4137, a H_2_S-slow releasing agent, rescued neonatal rat myocytes from hypertrophy induced by Ang-II and myocardial hypertrophy in spontaneously hypertensive rats, respectively [37,38]. The underlying mechanisms for H_2_S protection were attributed to two key proteins, Sirtuin 3 (SIRT3) and Krüppel-like factor 5 (KLF5). Sirtuin 3 (SIRT3) is closely associated with mitochondrial function and oxidative stress. NaHS increased SIRT3 promoter activity and SIRT3 expression in these cells and subsequently improved mitochondrial function and rescued the expression of FOXO3a and SOD2. The cardiac protective effects of NaHS were abolished by SIRT3 silencing. TAC-induced myocardial hypertrophy in wide-type mice was attenuated by NaHS, due to inhibited oxidative stress and improved mitochondrial ultrastructure. The expression of OPA1, MFN1 and MFN2 expression was upregulated but that of DRP1 and FIS1 downregulated [37]. On the other hand, hypertrophic myocardial samples from patients showed increased expression of KLF5. GYY4137 administration suppressed the over-expression of KLF5 in myocardium of spontaneously hypertensive rats and in hypertrophic rat neonatal cardiomyocytes. The altered expression of KLF5 was also implicated with the altered expression and activity of atrial natriuretic peptide expression and specificity protein 1. The pivotal roles of SIRT2 and KLF5 for the cardiac protective effects of H_2_S were further indicated since NaHS did not counteract myocardial hypertrophy in SIRT3 KO mice [37] and GYY4137 effect on neonatal rat cardiomyocytes was abolished by KLF5 knockdown [38]. The correlation of H_2_S-induced upregulation of SIRT3 and downregulation of KLF5 in the pathogenesis of myocardial hypertrophy is unclear. These two signaling cascades may be linked by H_2_S sequentially or parallelly. It is also likely that additional signaling events are regulated by H_2_S in this pathology.

Another pathogenic process leading to heart failure is myocardial fibrosis. Pan et al. [39] showed that H_2_S attenuated myocardial fibrosis by inhibiting NADPH oxidase-4 (Nox4) pathway and increasing HO-1 expression. Ang II activated cultured rat neonatal cardiac fibroblasts by upregulated Nox4 expression, which was abolished by NaHS. NaHS treatment also decreased ROS production and ERK1/2 phosphorylation and increased HO-1 expression. The observation made on cardiac fibroblasts was validated in a whole-animal myocardial infarction (MI) model. After the rat coronary artery was ligated, the ischemic myocardium exhibited fibrotic and inflammatory responses. These responses were reversed by NaHS treatment and the underlying mechanisms were also tracked back to H_2_S-triggered down regulation of Nox4 and upregulation of HO-1 and CSE.

### 3.2. The Neurobiological Targets of H_2_S

Abnormal H_2_S metabolism in the brain was related to oxidative neuronal damage and the pathogenesis of Alzheimer’s disease (AD). Tang XQ’s team at University of South China examined the beta-amyloid- and 1-methyl-4-phenylpyridinium ion-induced apoptosis of PC12 cells, a cell line derived from a pheochromocytoma of the rat adrenal medulla [40,41]. They found that NaHS protected PC12 cells against cytotoxicity and apoptosis by preserving mitochondrial function and boosting up the antioxidant protection. The neuronal protection offered by H_2_S at cellular level was confirmed by animal behavior. Tang XQ et al. [42] showed that intra-cerebroventricular injection of formaldehyde impairs the function of learning and memory of rats in the Morris water maze and novel object recognition test. With these cognitive damages, the expression of CBS in rat hippocampus was downregulated and the hippocampal production of H_2_S decreased. Although these were not lines of direct and complete evidence for the role of H_2_S in cognitive impairment or improvement, they nonetheless suggested the connection of H_2_S metabolism, oxidative stress, and brain cognitive functions. Furthermore, the antidepressant effects of H_2_S have been shown in various stress-induced animal models of depression, such as chronic unpredicted mild stress and chronic restraint stress [43,44].

H_2_S is also a gasotransmitter in the peripheral nervous system. Xu’s research team in Soochow University has contributed to our understanding of the role of H_2_S in chronic visceral hyperalgesia, inflammatory pain and neuropathic pain. During experimental chronical visceral hyperalgesia in rats, such as that occurred with irritable bowel syndrom, CBS expression was upregulated in colonic dorsal root ganglion (DRG) [45] due to p65 activation [46]. Inhibition of CBS activity with O-(Carboxymethyl)hydroxylamine hemihydrochloride (AOAA) or application of NaHS attenuated or enhanced neuronal excitability and potentiated sodium channel current densities of colon DRG neurons, respectively. The pro-hyperalgesia effect of H_2_S may be related to the expressional changes of multiple voltage-gated ion channels, such as the upregulation of sodium channel (Nav1.7 and Nav1.8) in DRGs [45,47] and inhibition of potassium channel (Kv1.1 and Kv1.4) [48] in trigeminal ganglion (TG) neurons. Another noticeable discovery make by Xu’s team is the epigenetical regulation of CBS expression by DNA demethylation of the promoter region of *cbs* gene [49].

### 3.3. H_2_S and Respiratory Diseases

Oxidative stress and inflammation constitute important pathogenic factors for chronic obstructive pulmonary disease (COPD). Most patients with COPD show a poor response to corticosteroids. The research team led by Dr. Chen YH at Peking University Third hospital is the first one to reveal the correlation of endogenous H_2_S in COPD airway inflammation and airway remodeling. This team observed that the serum H_2_S level is significantly higher in patients with stable COPD than in patients with acute exacerbation of chronic obstructive pulmonary disease (AECOPD) and age-matched control subjects. The serum H_2_S level was significantly lower in smokers than non-smokers, both populations constituted with AECOPD and healthy control subjects. The serum H_2_S level in patients with COPD is positively correlated with the percentage of forced expiratory volume in one second (FEV1), negatively correlated with the proportion of neutrophils in sputum, and positively correlated with the proportion of lymphocytes and macrophages [50].

The changes of exhaled H_2_S in COPD patients with different inflammatory phenotypes are correlated with exhaled NO. The exhaled level of H_2_S is higher in the non-eosinophilic cell group than in the eosinophilic cell group of COPD patients [51]. These studies showed that the endogenous H_2_S metabolic pathways in the lung tissue of patients with COPD have changed, and endogenous H_2_S can be used as a biomarker to reflect COPD airway inflammation and systemic inflammation.

Pathology studies on human peripheral lung tissue samples did not draw conclusion on COPD-related changes in expression of CSE and CBS. Immunohistochemistry showed that CSE was mainly expressed in bronchial and vascular SMCs and alveolar epithelial cells in non-smokers’ lung tissues. While the protein level of CSE was decreased in smokers and COPD patients in comparison with non-smokers, CSE mRNA level was downregulated. On the other hand, CBS mRNA level was lower in the lung tissues of smokers and COPD patients than in non-smokers. Quite contrary to the reported higher levels of H_2_S in serum and in exhaled air of COPD patients than healthy people, there was no significant difference in H_2_S levels in lung tissues from non-smokers, smokers, and COPD patients [52].

H_2_S as a therapeutic agent for bronchodilation was studied on chronic cigarette smoke-induced lung injury in rats. NaHS alleviated the airway reactivity induced by acetylcholine or potassium chloride, decreased lung pathology score, and inhibited IL-8 and TNF-α concentrations in lung tissues [53]. GYY4137 reduced the release of TNF-α and IL-8 induced by cigarette smoke in a concentration-dependent manner. In the alveolar macrophages of smoking rats, GYY4137 combined with dexamethasone reduced the concentration of TNF-α. These results demonstrate that H_2_S improves steroid sensitivity due to its anti-inflammatory and antioxidant effects [52]. Further research found that H_2_S played a protective role in particulate matter-induced mouse emphysema and airway inflammation by inhibiting NLRP3 inflammasome formation and apoptosis via Nrf2-dependent pathway [54].

### 3.4. Other Important Discoveries of H_2_S Biomedical Function

#### 3.4.1. Sulfur and Iron Interaction

H_2_S has significant effect on iron metabolism in mammals. This sulfur-iron linkage as well as its direct effect on heme synthesis and erythropoiesis is a seminal discovery made in China by Qian’s team. They have provided evidence that H_2_S acts on body iron homeostasis by regulating the expression of iron transport proteins via the interleukin-6 (IL-6)/signal transducer and activator of transcription 3STAT3/hepcidin pathway [55]. This conclusion was based on the effects of NaHS and GYY4137 on the expression of ferroportin-1 (Fpn1), transferrin receptor-1 (TfR1), hepcidin, IL-6 and pSTAT3 in the spleen of mice in vivo and peritoneal macrophage in vitro. In order to link changes in iron homeostasis with endogenous H_2_S, CBS knockout (KO) mice were examined. Anemia and iron overload in the serum, liver, spleen, and heart were noticed in CBS-KO mice with a hemochromatosis-like phenotype. Hepatic and serum hepcidin levels were high and iron usage by erythropoiesis were low, a phenomenon associated with IL-6 induced downregulation of erythropoietin. This iron-overload related phenotype was partially reversed by administration of CBS-overexpressing adenovirus into CBS mutant mice [56]. Qian’s team further showed that CBS deficiency significantly downregulated two key enzymes involved in the heme biosynthetic pathway, ALAS2 (delta-aminolevulinate synthase) and FECH (ferrochelatase). The expressions of EPO (erythropoietin), EPOR (erythropoietin receptor) and HIF-2á (hypoxia inducible factor-2 subunit á) in the blood, bone marrow or liver were also regulated by CBS. On the other hand, the expressions of IL-6 and hepcidin and iron content were increased in the blood, bone marrow or liver of CBS-KO mice. CBS deficiency-induced disruption in the expression of heme biosynthetic enzymes and heme-transporter results in the suppression of erythropoiesis [57]. Future studies are merited to determine whether the effect of CBS deficiency on iron overload and heme biosynthesis is actually due to deceased endogenous H_2_S level, considering that CBS deficiency would lead to dysregulated metabolism of homocysteine, L-cysteine, L-serine, á-ketobutyrate, NH_3_ as well as H_2_S [45]. This concern is validated by the study on CSE-KO mice [58]. Rather than anemia, CSE-KO mice exhibit elevated red blood cell counts and red blood cell mean corpuscular volumes compared to wild-type mice. Plasma and liver heme levels were elevated and coproporphyrinogen oxidase (CPOX), the sixth enzyme involved in heme biosynthesis, was upregulated in CSE-KO mice. In cells expressing a CPOX promoter construct system, H_2_S activated the CPOX promoter [58].

#### 3.4.2. Regulation of the Reproductive System by H_2_S

The first report on H_2_S metabolism in female reproduction system was made in 2009 when Patel et al. demonstrated the production of H_2_S in rat and human intrauterine tissues via CBS and CSE enzymes [59]. This H_2_S production was enhanced under hypoxia conditions. d’Emmanuele et al. showed that H_2_S mediated human corpus cavernosum smooth-muscle relaxation in 2009, constituting the first study on H_2_S metabolism and function in male reproduction system [60]. Multiple Chinse research teams have been studying the role of H_2_S in causing or treating erectile dysfunction [61,62,63,64]. The most original and impactful discovery made in China was a human study on sperm mobility and fertility [65]. Given that H_2_S is a potent anti-inflammatory gasotransmitter and antioxidant, H_2_S was hypothesized to be able to correct spermatogenic failure and testicular dysfunction. In this study, the researchers found decreased concentration of H_2_S in seminal plasma and down-regulated expression of CBS in sperm from both subfertile and infertile patients, especially asthenospermic patients. Human sperm motility was increased by exogenous H_2_S application to semen. Similar observations were obtained from lipopolysaccharide-treated mice, diabetic mice, and CBS-deficient mice. Not only this study outlines H_2_S metabolism in testicle and sperm, but also it suggests novel therapeutic strategy for impaired spermatogenesis and a defective blood-testis barrier.

## 4. Development of H_2_S-Based Novel Therapeutics

Translating fundamental research discovery into clinical practice and health management is the ultimate goal of biomedical research. This goal is especially relevant and reachable for H_2_S research. Numerous nutritional products, such as garlic, are rich sources for H_2_S supplement. Benavides et al. [66] showed that human red blood cells or rat aortic rings converted garlic-derived organic polysulfides into H_2_S, a process that relies on reduced thiols in or on cell membrane. H_2_S production from organic polysulfides is facilitated by allyl substituents and by increasing numbers of tethering sulfur atoms. Garlic-derived H_2_S caused vasorelaxation in vitro. This 2007 study inspired Zhu YZ’s team in China to search for ways to modify S-allyl-cysteine (SAC) isolated from garlic. Consequentially, a new H_2_S-releasing compound, S-propargyl-cysteine (SPRC), stable in the air, was developed [67]. SPRC (i.p.) was proved to attenuate cognitive impairment induced by intracerebroventricular injection of LPS in rats. LPS-induced drop in H_2_S levels in rat hippocampus was prevented by SPRC treatment. SPRC injection also inhibited LPS-induced neuroinflammation.

Another representative H_2_S-releasing compound synthesized by Chinese research teams is ZYZ-803 [68]. This H_2_S-NO hybrid slowly decomposes to release H_2_S and NO. It increased angiogenesis at the cellular, tissue, and animal levels in vitro and in vivo and its potency appeared to be greater than that of H_2_S and/or NO donor alone at the comparative concentration levels. Beyond directly releasing H_2_S and NO from the compound, ZYZ-803 also stimulated H_2_S production from CSE and NO production from eNOS. An Sirtuin-1 (SIRT1)/VEGF/cGMP signaling cascade is believed to mediate the proangiogenic effect of ZYZ-803.

The effectiveness and applicability of SPRC and ZYZ-803, in comparison with other well-developed H_2_S donors, merit further investigation. Their recognition and examination by other research teams in the world will also help these novel compounds gaining wider appreciation.

Table 2 lists some leading H_2_S researchers in China and their major contributions to this field that are most innovative and impactful. 

## 5. Foresight

**Figure 4:**
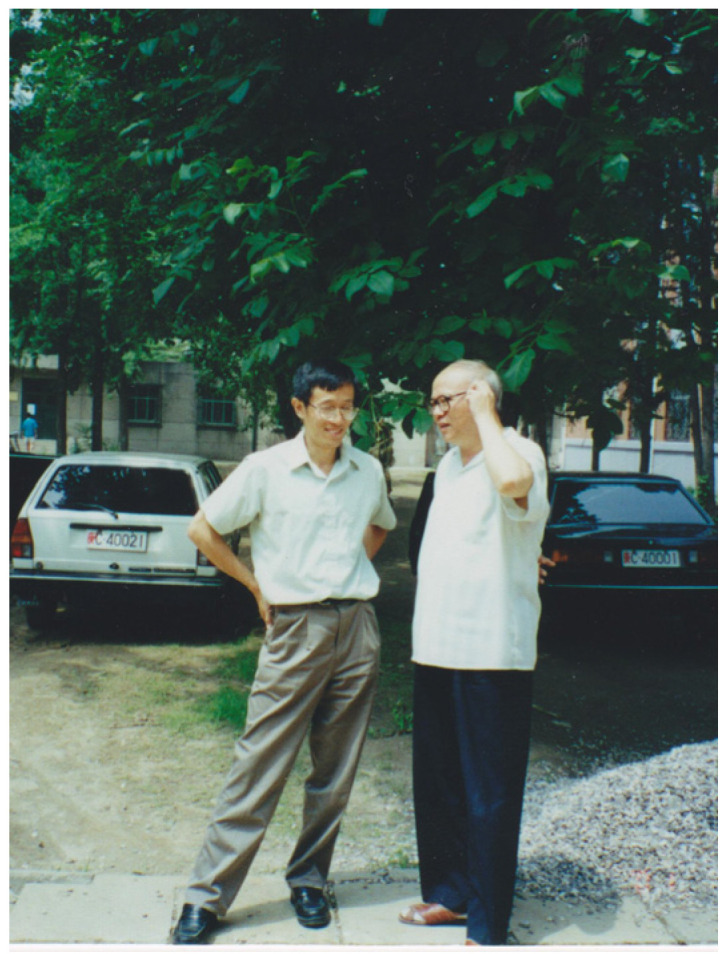
Brainstorm discussion of the authors in 2001 in Beijing, China.

H_2_S biomedical research in China has seen two decades of ground-breaking achievement and progress. Interest and attention to this field have never been this high. Research talents have been assembled in numerous post-secondary institutions and research organizations. Increased national funding and strengthened national technology platforms fuel the field and inject enthusiasm. When the authors of this article met in 2001 to brainstorm the trend and direction of biomedical research on small gaseous molecules in China and in the world, we were excited about the potential and impact of this research field (Figure 4). We have every reason to believe that the momentum built up so far will be sustained over the next two decades. Pivoting from hindsight to foresight, the following suggestions are made as food for thought.

- As a nation, China needs to have a strategic and systemic plan for efficient and accelerated advancement of H_2_S biomedical research. As the organizations, China universities and research institutes need to identify and strategically support H_2_S biomedical research clusters to extend the frontiers of knowledge.
- National and international collaboration and coordinated and networking effort should be continued and strengthened. Persistent but not momentary in the fashion, and organized but not spontaneous or sporadic, are the formula of success for bigger bloom of H_2_S biomedical research in China.
- Ground-breaking fundamental H_2_S biomedical research, rather than patching and catching studies, should be encouraged and supported. National and institutional scientific goals in this field should be in place to assure the resource sufficient and metrics laid out.
- Knowledge translation efforts from bench to bedside should be identified and supported. It offers the most tangible benefits to human beings by targeting at the most relevant diseases with high morbidity and mortality/prevalence which are closely related to H_2_S metabolism and functions.
- Specifically designed training programs should be launched for next generation of H_2_S scientists, graduate students, post-doc fellows and young scientists.
- H_2_S biomedical research in China should seek integration and collaboration with other disciplines, such as physics, chemistry, engineering, agriculture, plant science, natural resource management, nutrition, etc.
- The immersion of H_2_S biomedical study with the Traditional Chinese Medicine (TCM) should be explored. H_2_S as well as other gasotransmitters may underly the chemical nature of “Qi” in TCM, of which the straight translation is “gas”. From this lens, intangible Qi would become tangible gasotransmitters [84]. As acupuncture in TCM may stimulate the balance and flow of Qi, whether H_2_S metabolism is regulated by acupuncture becomes intriguing. Furthermore, many Chinese herbs are known to affect microbial production of H_2_S, such as Dang-shen (Codonopsis pilosula) [85] and Gan-cao (Licorice) [86]. It would be of significant importance to explore the use of Chinese medicinal herbs for providing exogenous source of H_2_S and for regulating endogenous H_2_S metabolism.

“We are what we smell” [87]. With a comprehensive understanding of the physiological and pathophysiological importance of H_2_S, we can make our lives Healthier, Happier, and Sustained–one of the connotations of H_2_S.

## Figures and Tables

**Table 1:** Major conferences on H_2_S biology and medicine held in China since 2002.

Conferences	Date	Location	Attendees	Organizers
The 1st China National symposium on H_2_S gasotransmitter	24 November 2007	Beijing, China	169	Tang C, Du J, Zhu YC, Zhu YZ, Yao T
The First International Conference of H_2_S in Biology and Medicine	26–28 June 2009	Shanghai, China	~250	Wang R (Canada), Zhu YZ (China)
The 2nd China National symposium on H_2_S gasotransmitter	26 June 2010	Beijing, China	150	Du J, Tang C, Zhu YC, Zhu YZ
The 3rd China National symposium on H_2_S gasotransmitter	26 October 2013	Beijing, China	200	Tang C, Du J, Zhu YC, Zhu YZ, Jin H, Geng B

**Table 2:** Major discoveries on H_2_S biomedical research made by scientists affiliated with Chinese institutions.

Name *	Affiliation	Discoveries	References	Future Research Directions
Chen, Yahong	Peking University, Third Hospital	⮚ Correlation of abnormal H_2_S metabolism with stable COPD patients Endogenous H_2_S level as a biomarker of COPD airway inflammation; and exogenous H_2_S as a therapeutic agent for bronchodilation.	❖ Chen YH et al., 2005 [50] Zhang J et al., 2014 [51] Sun Y et al., 2015 [52] Chen YH et al., 2011 [53]	The mechanism of H_2_S effects on COPD. Treatment and prevention of COPD with H_2_S-predrugs.
Geng, Bin	Chinese Academy of Medical Sciences and Peking Union Medical College,	⮚ Endogenous production of H_2_S in adipose tissues and its vasorelaxant effect. H_2_S lowered hyper-homocysteinemia, protecting the cardiovascular system.	❖ Fang L et al., 2009 [69] Fan J et al., 2019 [70]	The regulatory role of CSE/H_2_S in perivascular diseases and the underlying immunological and epigenetic mechanisms.
Ji, Yong	Nanjing Medical University	⮚ Applications of GYY4137 to atherosclerosis, myocardial hypertrophy, and myocardial ischemia reperfusion injury. H_2_S prevents spermatogenic failure and testicular dysfunction. H_2_S-induced S-sulfhydration of Keap1, c-Jun, and specificity protein 1.	❖ Meng G et al., 2018 [37] Wang J et al., 2018 [65] Xie L et al., 2016 [33]	Regulatory mechanism for H_2_S production in different cells and diseases. Altered functions of H_2_S in aortic dissection, aortic aneurysm, etc. Therapeutic potential of H_2_S donors.
Li, Hongzhu	Xiamen University	⮚ H_2_S protects the heart from ischemic post-conditioning by increasing autophagy and upregulating HB-EGF/EGFR signaling.	❖ Chen J et al., 2016 [71] Zhang Y et al., 2019 [72]	Interaction of CSE/H_2_S with dopamine D1 receptors in diabetic vascular SMCs. Interaction of H_2_S with Hrd1-NLRP3- pyroptosis in cardiomyocytes senescence.
Tang, Chao-Shu Du, Junbao Jin, Hong-Fang	Peking University	⮚ Altered H_2_S metabolism with various types of hypertension and shock. The role of endogenous H_2_S in the development of atherosclerosis. The role of endogenous H_2_S in the development of various types of pulmonary hypertension and pulmonary structural remodeling. H_2_S controls inflammatory response and the molecular mechanisms. H_2_S inhibits myocardial damage and the mechanisms The inhibitory effect of H_2_S on vascular smooth muscle cell proliferation, involving the inhibition of MAPK pathway. A H_2_S-dependent region-specific chromatin remodeling of MAPKs pathway-associated genes for the regulation of cell growth and proliferation.	❖ Du J et al., 2002 [73] Jin HF et al., 2008 [74] Zhong G et al., 2003 [15] Wang Y et al., 2009 [32] Li L et al., 2013 [31] Du J et al., 2014 [35] Feng S et al., 2017 [19] Huang P et al., 2015 [16] Jin H et al., 2006 [20] Tian X et al., 2021 [22] Wei H et al., 2010 [36] Zhang D et al., 2019 [21] Zhang Q et al., 2004 [18] Zong Y et al., 2015 [17]	The interaction between H_2_S and sulfur dioxide (SO2) and its physiological and pathophysiological significance; The pathophysiological significance of the sulfur-containing gases and the therapeutic targets in cardiovascular diseases.
Tang, Xiao Qing	University of South China	⮚ The anti-depressant effects of H_2_S in various stress-induced animal models of depression. The protective effect of H_2_S against neuronal cytotoxicity.	❖ Liu HY et al., 2020 [44] Wei L et al., 2018 [43] Tang XQ et al., 2013 [42] Tang XQ et al. 2008 [40]	The mechanisms underlying H_2_S-enhanced warburg effect in the hippocampus H_2_S-regulated immunometabolism.
Wu, Yuming	Hebei Medical University	⮚ Decreased CSE expression in renal arteries from renal hypertension objectives; and decreased CBS expression in RVLM from spontaneous hypertensive rats. Various neuronal signaling pathways involved in the anti-hypertensive effects of H_2_S.	❖ Tian DY et al., 2017 [26] Xiao L et al., 2018 [25] Teng X et al., 2019 [27] Duan XC et al., 2015 [28]	The mechanisms underlying the inhibitory effects of H_2_S on sympathetic overactivity in hypertension.
Xu, Guang-Yin	Soochow University	⮚ Chronic visceral hyperalgesia is associated with upregulation of CBS in colonic DRG. CBS-H_2_S signaling regulated the expression and function of multiple voltage-gated ion channels in peripheral neurons.	❖ Qu R et al., 2013 [75] Hu S et al., 2013 [47] Wang Y et al., 2012 [45]	The origins and roles of H_2_S in chronic visceral hyperalgesia Novel H_2_S-based therapeutical strategies.
Zhang, Weihua	Harbin Medical University	⮚ Decreased H_2_S level and increased ubiquitination of cardiac structural proteins, such as MYH6 and MyL2 in diabetic cardiomyopathy. H_2_S switched cardiac energy substrate utilization from fatty acid oxidation to glucose.	❖ Sun Y et al., 2018 [76] Sun X et al., 2020 [77] Yu M et al., 2020 [78]	H_2_S regulation of lipid droplets accumulation in diabetic hearts
Zhu, Yi-Chun	Fudan University	⮚ H_2_S promoted angiogenesis in vitro and in vivo. The molecular interaction between H_2_S and receptor tyrosine kinases (RTKs) family with the common involvement of disulfide bonds.	❖ Cai WJ et al., 2007 [11] Tao BB et al., 2013 [79] Xue R et al., 2013 [80] Ge SN et al., 2014 [81]	To validate the hypothesis that H_2_S targets some common motif contained in various proteins, based on the locations and functions of disulfide bonds.
Zhu, Yi-Zhun	Marco Science and Technology University	⮚ H_2_S-ameliorated mitochondrial dysfunction in the cardiovascular system. Invention of novel H_2_S-releasing compound S-propargyl-cysteine (SPRC), and H_2_S-NO hybrid ZYZ-803.	❖ Wang Q et al., 2010 [82] Wu W et al., 2019 [83] Gong QH et al., 2011 [67] Hu Q et al., 2016 [68]	The mechanisms and therapeutic potencies of novel H_2_S donors in diseases such as rheumatoid arthritis, respiratory diseases, and coronavirus disease.

* The first column follows the last name in alphabetical order.
